# Effect of Ionic Liquids on the Structural Properties of SBA-15/CeO_2_ Nanocomposites

**DOI:** 10.1021/acsomega.5c01826

**Published:** 2025-05-27

**Authors:** Danilo W. Losito, Renato M. Latini, Norberto S. Gonçalves, Fernanda F. Camilo, Márcia C. A. Fantini, Tereza S. Martins

**Affiliations:** † Departamento de Química, Instituto de Ciências Ambientais, Químicas e Farmacêuticas, Universidade Federal de São Paulo, Diadema 09913-030, SP, Brazil; ‡ Laboratório de Cristalografia, Instituto de Física, Universidade de São Paulo, São Paulo 05508-090, SP, Brazil

## Abstract

Ordered mesoporous silica materials have attracted considerable attention due to their unique structural, textural, and morphological properties, alongside their versatile applications in catalysis, adsorption, and drug delivery systems. This study explores the influence of ionic liquids (ILs) on the synthesis of SBA-15 and SBA-15/CeO_2_ nanocomposites. DMIBr (1-dodecyl-3-methylimidazolium bromide) and DMIBF_4_(1-dodecyl-3-methylimidazolium tetrafluoroborate) were the analyzed ILs. The objective was to evaluate how these ILs modulate the structural, textural, and morphological properties of the resulting nanocomposites. SAXS analysis confirmed the formation of well-ordered mesoporous structures with hexagonal arrangements, corroborating with physisorption data and TEM images. XRD measurements confirmed the presence of CeO_2_ nanoparticles within the nanocomposites, exhibiting a fluorite-type cubic crystal structure, supported by Raman spectroscopy, which identified characteristic peaks corresponding to Ce–O vibrational modes. XPS analysis provided detailed insights into the surface chemistry, revealing the presence of Ce^3+^ and Ce^4+^ ions, oxygen species, and their interaction with the silica matrix. The Ce^3+^ ions, associated with oxygen vacancies in the CeO_2_ lattice, were identified as key active sites for adsorption and catalysis. SEM images displayed distinct morphologies of the nanocomposites, attributed to the specific used IL. These results underscore the pivotal role of ILs in tailoring the properties of SBA-15/CeO_2_ nanocomposites, offering valuable knowledge for their potential applications in diverse fields.

## Graphical abstract

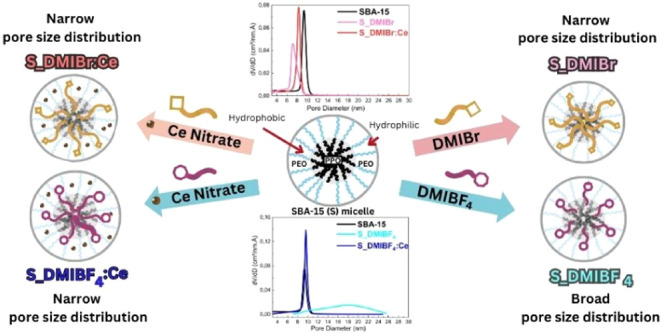

## 1 Introduction

The critical challenges posed by global warming and water pollution necessitate the development of sustainable technologies for environmental applications.^1–4^ Some of these technologies require specially designed materials for CO_2_ capture and storage,^5–7^ surface modification,^8–11^ adsorption and degradation of organic dye pollutants,^12,13^ among others.^14,15^ A promising material for these applications is cerium oxide (CeO_2_), which has demonstrated high efficiency in environmental management, playing a crucial role in carbon dioxide capture and organic pollutant degradation.^16,17^

Integrating cerium oxide into mesoporous silica enhances catalytic and adsorptive capabilities, making it highly beneficial for various industrial applications. The combination promotes a greater dispersion of CeO_2_ particles and facilitates access to catalytic sites, significantly boosting catalytic activities, particularly in oxidation–reduction reactions.^18,19^ The well-ordered framework of mesoporous silica improves adsorption capacity, enabling the efficient removal of pollutants such as heavy metals and organic compounds from water.^20–22^ Furthermore, the synergistic effects between CeO_2_ and other catalytic metals, well dispersed within the mesoporous silica structure, enhance the overall performance of the composite, optimizing electron transfer processes, which are crucial for catalytic activity.^23,24^

SBA-15, an ordered mesoporous silica, is an exceptional support for CeO_2_ due to its unique properties. It features a high surface area (∼800 m^2^g^–1^), a pore volume ranging from 1 to 5 cm^3^g^–1^, an adjustable pore size (5–30 nm), and remarkable thermal and mechanical stability. These characteristics make it highly suitable for pollutant adsorption and degradation.^22,25^ Furthermore, its mesoporous structure, rich in silanol groups, facilitates the incorporation of inorganic species, such as CeO_2_ nanoparticles, thereby enhancing the stability of the supported material, a crucial feature for environmental applications.^26–30^ Additionally, the possibility of tuning the textural, morphological, and structural properties of SBA-15 through the controlled synthesis parameters further underscores its versatility in sustainable technologies.^31–33^

Ionic liquids are gaining attention as effective additives in synthesizing nanomaterials and SBA-15 composites. These substances offer several advantages: they are thermally stable and possess negligible vapor pressure, which makes them excellent solvents for high-temperature reactions without the risk of evaporation.^34^ Additionally, ionic liquids’ structural versatility allows the customization of their ionic structures to tailor specific interactions with target materials.^34–38^ This adaptability can lead to better control over nanoparticle size, shape, and distribution within the composite material.^39,40^ Moreover, ionic liquids can facilitate the formation of well-defined pore structures and enhanced porosity in ordered mesoporous materials, like SBA-15, contributing to increased surface areas and potentially improving the material’s catalytic and adsorption capabilities.^41–43^ Their ability to act as templating agents also promotes changes in metal oxide nanoparticles’ structural and morphological properties.^44–46^ The presence of IL in the reaction medium leads to high nucleation rates and, consequently, to smaller particles, playing an essential role in the catalytic properties.^47–50^

In this context, Jardim et al.^31^ explored the influence of different ionic liquids in preparing SBA-15/TiO_2_ nanocomposites. They utilized a one-step process where ionic liquids served as agents to control the crystalline phases of TiO_2_ particles embedded in the SBA-15 matrix. Their findings indicated that the ionic liquid 1-hexadecyl-3-methylimidazolium tetrafluoroborate significantly promotes the formation of the anatase phase of TiO_2_. Conversely, 1-hexadecyl-3-methylimidazolium bis­(trifluoromethanesulfonyl)­imide tends to favor the rutile phase, but this effect becomes noticeable only at higher concentrations of the ionic liquid.

Regarding the influence of ionic liquids in the synthesis of mesoporous materials, several studies have been reported using imidazolium-based IL (1-alkyl-3-methylimidazolium - C*_n_* MI, where *n* is the number of carbons in the alkyl chain) as a template to provide different particle morphologies (size and shape).^51–54^ Trewyn et al.^55^ reported the use of 1-tetradecyl-3-methylimidazolium bromide (C_14_MIMBr), 1-hexadecyl-3-methylimidazolium bromide (C_16_MIMBr), 1-octadecyl-3-methylimidazolium bromide (C_18_MIMBr) in the MCM-41 synthesis. As a result, each IL gave different particle morphology, pore size, and surface area, showing the influence of diverse alkyl chains on the formation of the MCM-41 structure. In another report, Wang et al.^52^ also showed the influence of 1-hexadecyl-3-methylimidazolium chloride in the synthesis of MCM-41 and MCM-48.

Although some studies highlight the key role of IL in the synthesis of mesoporous materials, such as MCM type and oxide nanoparticles, there are few studies considering its use in the synthesis of SBA-15 mesostructured material and in the synthesis of nanocomposites of SBA-15/CeO_2_.

In this context, this study explores the use of different ionic liquids in synthesizing SBA-15:CeO_2_ nanocomposites. Two distinct ionic liquids were employed (Figure 1): DMIBr (1-dodecyl-3-methyl imidazolium bromide) and DMIBF_4_ (1-dodecyl-3-methyl imidazolium tetrafluoroborate) to investigate how variations in anions (bromide vs tetrafluoroborate) affect the preparation, characteristics, and performance of the materials. The primary objective is to assess how these ionic liquids influence the structural and morphological characteristics of CeO_2_ combined into the SBA-15 matrix. The materials developed through this research have potential applications in various segments, including environmental remediation, where they can be used for pollutant removal from water and air. It is worth mentioning that no study in literature focuses explicitly on the influence of ionic liquids over the structural and morphological characteristics of CeO_2_ combined with SBA-15, underscoring the contribution of our research in this area.

**Figure 1:**
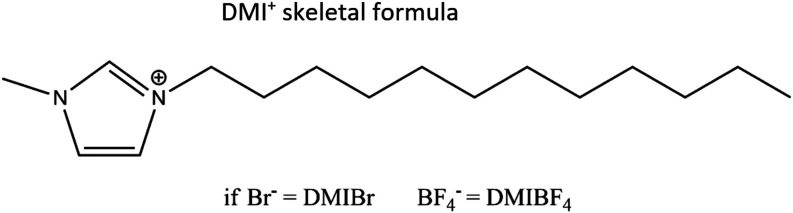
Representation of the ionic liquid structures. The alkyl chain corresponds to the DMI^+^ skeletal formula. If the anion is Br^–^ (bromide ion), the compound is DMIBr; if the anion is BF_4_^–^ (tetrafluoroborate ion), the compound is DMIBF_4_.

## 2 Experimental Section

### 2.1 Materials

All chemicals were purchased and used as received. Tetraethylorthosilicate (TEOS, 98%, Sigma-Aldrich, Brazil), nonionic triblock copolymer surfactant, Pluronic P123 [(poly­(ethylene oxide)-poly­(propylene oxide)-poly­(ethylene oxide), (EO_20_PO_70_EO_20_), Sigma-Aldrich, Brazil], hydrochloric acid (HCl, 37%, Synth, São Paulo, Brazil), cerium­(III) nitrate hexahydrate (99,99%, Sigma-Aldrich, Brazil). SBA-15 was prepared following the methodology described by Zhao et al.^56^

### 2.2 Ionic Liquids Preparation

Both ionic liquids were prepared according to the procedures described in the existing literature.^31,57^

### 2.3 CeO_2_–SBA-15 Nanocomposites Preparation

First, SBA-15 modified with the ionic liquids (referred to as S_IL) samples were prepared using 2.00 g of Pluronic P123 dissolved in 75 mL of HCl 1.6 mol L^–1^, followed by adding 0.70 mmol of each ionic liquid. Afterward, 4.45 mL of TEOS were added, and the mixture was stirred for 24 h at 40 °C, followed by hydrothermal treatment in a Teflon-lined autoclave at 100 °C for 48 h. The material was stirred at 80 °C until the complete elimination of the solvent and then calcinated at 540 °C, using a heating rate of 2 °C min^–1^ in an air atmosphere. After reaching this temperature, it was kept under the same conditions for 3 h to eliminate the carbonaceous material.

The SBA-15_IL:CeO_2_ composites were prepared following the methodology described by Jardim et al.^31^ with some modifications. Succinctly, SBA-15:IL:CeO_2_ samples were prepared using the following procedure: 0.927g of Ce­(NO_3_)_3_·6H_2_O, 0.7 mmol of each ionic liquid, and 4.45 mL de TEOS were added simultaneously to the Pluronic P123 (75 mL of HCl 1.6 mol·L^–1^). The hydrothermal treatment, solvent elimination, and calcination steps were performed similarly to those described for the SBA-15IL samples (S_IL). The resulting materials exhibited molar ratios of CeO_2_ to SBA-15 and IL to SBA-15 of 10% and 0.035%, respectively. These samples are denoted as S_IL:Ce, where S refers to SBA-15, IL to the ionic liquid used, and Ce indicates the presence of CeO_2_.

SBA-15:CeO_2_ nanocomposite in the absence of ionic liquids was prepared by direct synthesis for comparison. For this preparation, 0.93 g of Ce­(NO_3_)_3_·6H_2_O and 4.45 mL de TEOS were added simultaneously to the Pluronic P123 (75 mL of HCl 1.6 mol·L^–1^). The hydrothermal treatment, solvent elimination, and calcination steps were performed similarly to the previous synthesis.

The quantities of the reactants used to synthesize the materials described above are detailed in Table S1 of the Supporting Information.

### 2.4 Characterization

Small-angle X-ray scattering (SAXS) curves were acquired on a Nanostar (Bruker) instrument, with a point beam generated by a conventional copper tube (K_α_, Cu = 0.15418 nm), a current of 30 mA, and an accelerated tension of 40 kV. The setup utilized Göbel mirror geometry and a bidimensional detector, with 64.8 cm (0.2 ≤ *q* ≤ 3.5 nm^–1^) between the sample and detector.

Scanning electron microscopy (SEM) images were acquired using a JSM-6610LV (JEOL) instrument operating with a secondary electron imaging (SEI) detector. Prior to measurement, samples were placed onto conductive double-sided adhesive carbon tape and coated with a thin layer of gold. Transmission electron microscopy (TEM) images were acquired using a JEOL JEM 2100 microscope equipped with an energy-dispersive X-ray spectrometer (EDS). A drop of the sample, dispersed in water, was placed onto a carbon-coated copper (Cu) grid and dried at room temperature.

Nitrogen adsorption/desorption isotherms (NAI) were recorded on a NOVA 2 porosimeter (Micromeritics - ASAP 2020), with degassing at 200 °C (until vacuum ≤ 10 μm Hg) for approximately 12 h. The specific surface areas of mesoporous and microporous were calculated through the BET (Brunauer-Emmet-Teller) method^58^ and the t method^59^ (”t-plot”), respectively. Pore size distribution and cumulative pore volume were determined using the BJH (Barrett–Joyner–Halenda) method^60^ of adsorption.

The crystallinity of the materials was evaluated by XRD (Powder X-ray Diffraction) and Raman spectroscopy. X-ray diffractograms were obtained using an Ultima+ (Rigaku) equipment, with Cu Kα radiation (λ = 0.15418 nm) and the powder method in a θ-θ geometry. The selected angular range was from 5 to 90° (2θ), with a step size of 0.05°, a counting time of 2.0 s, operating at 40 kV, 30 mA, with sample support rotating at 30 rpm. Raman Spectroscopy data were obtained using a Raman Renishaw microscope, model InVia, equipped with a CCD multichannel detector, He–Ne lasers (632.8 nm), and diode lasers (830 nm). The spectra had a spectral resolution of 4 cm^–1^, obtained through three accumulations, an integration time of 20 s, and a spectral range from 100 to 2000 cm^–1^.

Fourier transform infrared (FTIR) spectra were recorded in the range 4000–400 cm^–1^, using an Agilent Cary 630 FTIR spectrometer. IR measurements were performed in attenuated total reflection (ATR) mode.

Thermogravimetric analysis (TGA) and Differential Scanning Calorimetry (DSC) measurements were conducted using a Discovery SDT 650 Simultaneous Thermal Analyzer DSC/TGA system from TA Instruments. The data were obtained at a heating rate of 10 °C min^–1^, within a temperature range from 35 to 900 °C, under a dynamic air atmosphere (100 mL min^–1^), employing an alumina crucible (90 μL) with approximately 5 mg of the sample mass.

X-ray photoelectron spectra (XPS) were obtained at LNNano-CNPEM, Brazil, with a Thermo Scientific K-Alpha X-ray Photoelectron Spectrometer System using a monochromatic Al K-Alpha (1486.6 eV) source. The analysis was performed with 10 scans in a spot size of 300 μm, a pass energy of 50.0 eV, an energy step size of 0.10 eV, and a dwell time of 50 ms. The as-obtained data were analyzed using Casa XPS software (version 2.1.0.1).

## 3 Results and Discussion

The synthesis of SBA-15:CeO_2_ nanocomposites was conducted using two distinct ionic liquids (ILs): 1-dodecyl-3-methylimidazolium bromide (DMIBr) and 1-dodecyl-3-methylimidazolium tetrafluoroborate (DMIBF_4_). As mentioned in the introduction, ILs can facilitate the formation of well-defined pore structures and enhance porosity, contributing to increased surface areas and improved catalytic and adsorption capabilities. The choice of ILs with different anions bromide (Br^–^) and tetrafluoroborate (BF_4_^–^) was strategic to investigate the anion effect on the synthesis and properties of the nanocomposites. The Br^–^ ion, being a halide with strong coordinating ability, may interact differently with Ce^3+^ ions compared to the BF_4_^–^ ion, which is a larger, more weakly coordinating anion. These interactions can modify the nucleation and growth processes of CeO_2_ nanoparticles within the SBA-15 matrix. For instance, the presence of ILs in the reaction medium can also lead to higher nucleation rates, resulting in smaller particle sizes and potentially enhancing catalytic properties. The choice of IL cations with long alkyl chains (DMI) could influence the micelle formation of Pluronic P123, leading to variations in pore size and wall thickness of the mesoporous silica. To evaluate the impact of ILs on the structural and morphological characteristics of the nanocomposites, various characterization techniques were used. For comparison, SBA-15 (referred to as S:IL) was also prepared in both ionic liquids, and SBA-15:CeO_2_ without ionic liquid (referred to as SBA-15:CeO_2_) was obtained by direct synthesis.

All SAXS curves shown in Figure 2 exhibit the five typical peaks of SBA-15, indexed as the (100), (110), (200), (210), and (300) reflections. This confirms that all composites prepared with both ILs do not disrupt the two-dimensional hexagonal structure with space group *p6 mm* characteristic of SBA-15.^31,32,61^ The lattice parameters (*a*_(hkl)_) and interplanar distances (*d*_(hkl)_) for all samples are summarized in Table 1. For all materials, these peaks shifted to higher *q* values­(Å^–1^) compared to SBA-15, indicating lower values for *d*_(hkl)_ and *a*_(hkl)_. This result can be explained by the hydrophilic/hydrophobic characteristics of the ILS, which may contribute to an overall smaller pore size after calcination. Moreover, S_IL prepared in DMIBr shows a more pronounced change, exhibiting smaller lattice parameters and interplanar distances than samples prepared with BF_4_^–^ ionic liquid. When comparing materials prepared in DMIBr versus DMIBF_4_, the peaks indicating the two-dimensional mesoporous structure are more intense and better defined for the sample prepared with the Br-based IL. Possibly, the smaller anion causes less disruption to the SBA-15 mesophase, resulting in more orderly arranged mesopores.

**Figure 2:**
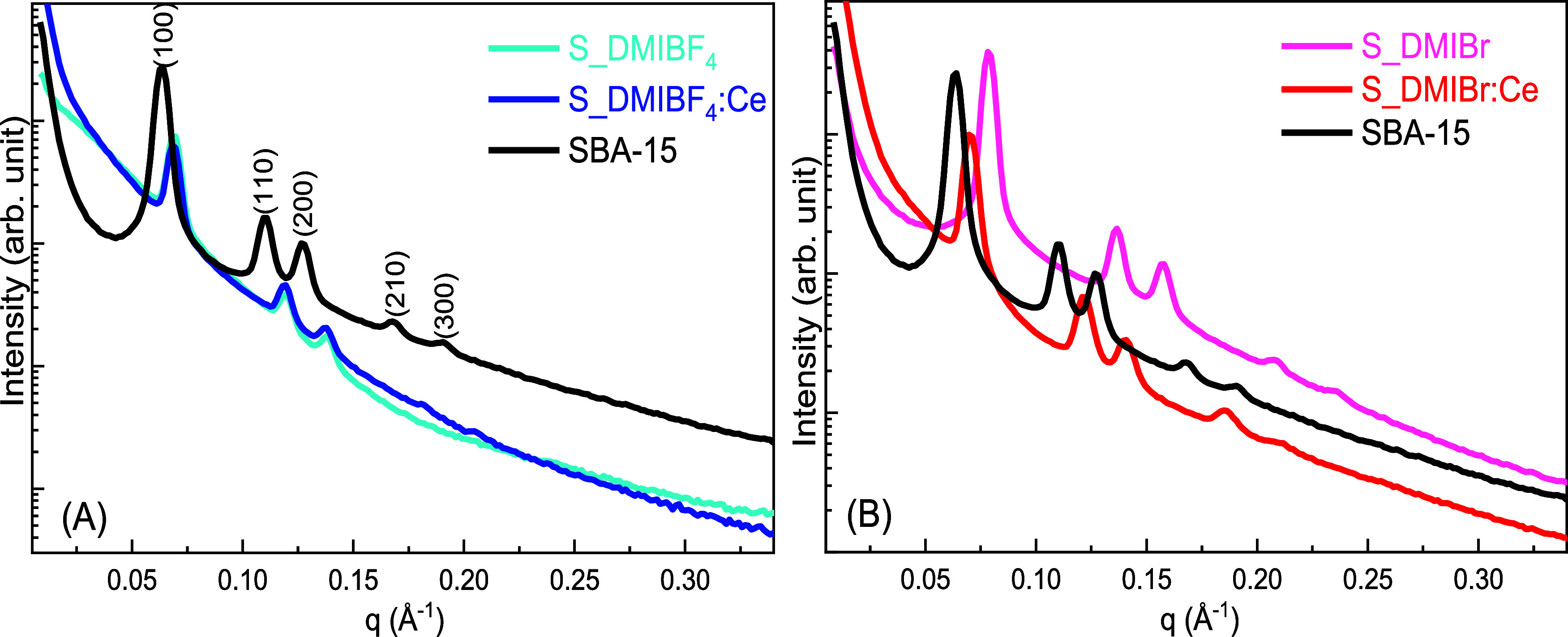
SAXS curves of the prepared materials: (A) SBA-15, S_DMIBF_4_, and S_DMIBF_4_:Ce, (B) SBA-15, S_DMIBr, and S_DMIBr:Ce.

**Table 1:** Structural Properties of SBA-15 and SBA-15/CeO_2_ Nanocomposites Prepared in Ionic Liquid, Obtained from SAXS Analysis^a^

	*d*_(hkl)_/*a*_(hkl)_ (nm)				
samples	(100)	(110)	(200)	(210)	(300)
**SBA-15**	9.9/11.4	5.7/11.3	4.9/11.4	3.7/11.4	3.3/11.4
S_DMIBr	8.0/9.2	4.6/9.2	4.0/9.2	3.0/9.2	2.6/9.2
S_DMIBF_4_	9.1/10.5	5.3/10.5	4.5/10.5		
S_DMIBr:Ce	8.9/10.3	5.1/10.3	4.5/10.3	3.4/10.3	3.0/10.3
S_DMIBF_4_:Ce	9.2/10.6	5.3/10.5	4.6/10.5	3.5/10.6	

a *a*_(hkl)_ = lattice parameter; *d*_(hkl)_ = interplanar distance; The error of the lattice parameter is 2%.

Comparing S_IL and S_IL:Ce samples prepared in DMIBr (Figure 1B), it is noted that the presence of cerium slightly increases the lattice parameters (*a*_(hkl)_). This effect can be explained by the “salting-in” effect caused by the Ce­(NO_3_)_3_·6H_2_O precursor, which expands the pores during the electrostatic interaction between the Pluronic P123 and the ion in solution.^62,63^ However, this effect is not observed in the sample prepared with the BF_4_^–^ anion, probably due to the larger size of the BF_4_^–^ ion, which diminishes the ’salting-in’ effect. The comparison between samples prepared with and without IL highlights the crucial role of ionic liquids in stabilizing the SBA-15 mesostructure during synthesis. While the samples prepared with ILs retain the characteristic hexagonal mesostructure, the sample prepared without IL (S:Ce) shows a collapsed structure, as evidenced by the absence of the typical five peaks of SBA-15 (Figure 1S). This suggests that ILs act as cotemplates, interacting with the mesophase and also by mitigating the disruptive ″salting-in″ effect of cerium nitrate during calcination.

XRD measurements were performed to verify the crystalline phase of cerium oxide in SBA-15:Ce nanocomposites prepared in the different ionic liquids (Figure 3). All prepared samples displayed diffraction peaks at (111), (200), (220), (311), (222), (400), (331), (420), (422), confirming the presence of pure cubic fluorite cerium oxide with *Fm3m* space group, as the sample S:Ce prepared in the absence of ionic liquid (Figure 2).^64,65^ All nanocomposites exhibited well-defined peaks, indicating a highly crystalline structure for CeO_2_. The crystallite sizes for all samples were found to be very similar, ranging from 20.3 to 21.4 nm, calculated by the well-known Scherrer equation. This observation suggests that the type of anion in the ionic liquids does not significantly influence the crystallite size of CeO_2_ in the SBA-15 nanocomposites. The results show that while different ionic liquids, present in the synthesis of SBA-15, interfere in the mesostructure of the ordered porous matrix, but maintaining the two-dimension hexagonal arrangement, the type of anion in the ionic liquid does not significantly affect the crystallite size (Table 2) of cerium oxide within the nanocomposites. The uniformity in crystallite sizes suggests that the confinement by the SBA-15 framework is the predominant influence on crystallite growth. Due to the fact that the mesopore size is around 10 nm or less in its entrance, and the CeO_2_ crystallites have a dimension of ∼20 nm, it is reasonable to state that these crystallites are located in the macroporous region.

**Figure 3:**
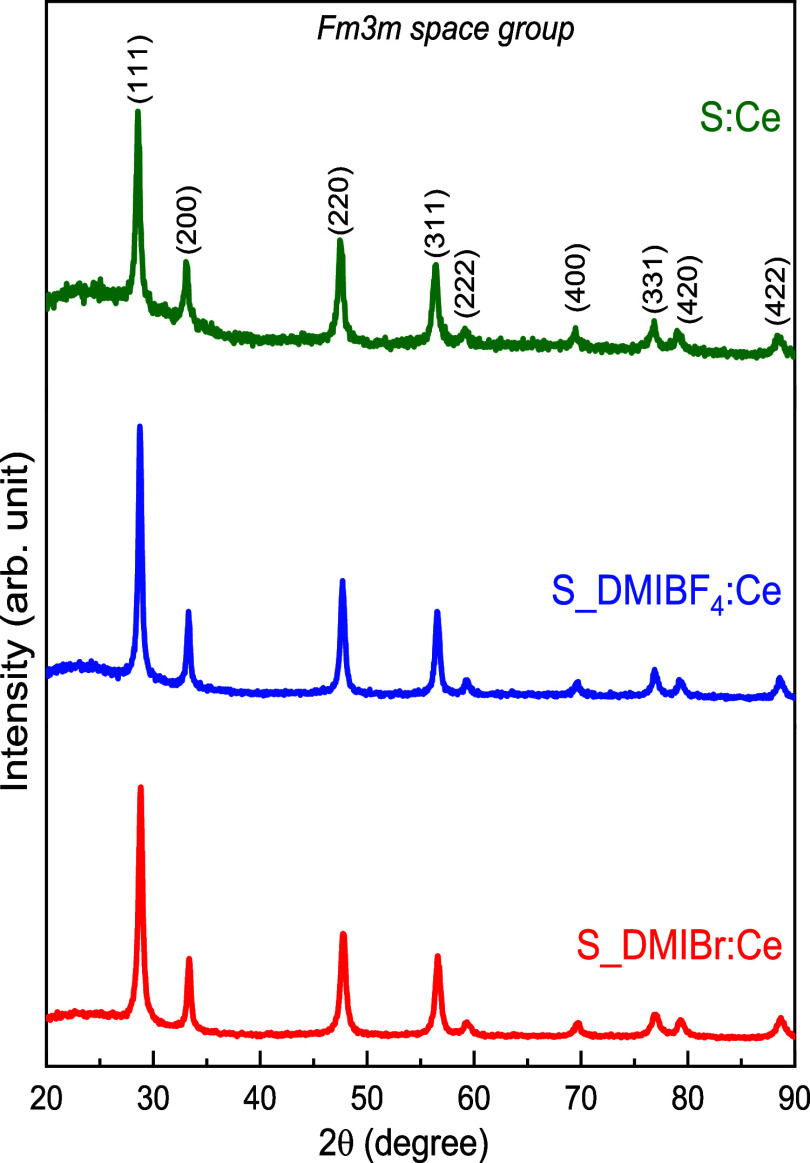
XRD patterns of S:Ce and S_IL:Ce nanocomposites (S_DMIBr:Ce and S_DMIBF_4_:Ce).

**Table 2:** CeO_2_ Crystallite Size and Raman Peak Data of SBA-15/CeO_2_ Nanocomposites Prepared with Different Ionic Liquids

sample	crystallite size (nm)	integrated area Raman peak (arb. unity)
S_DMIBr:Ce	20.3	80,142
S_DMIBF_4_:Ce	21.2	284,802
S:Ce	21.4	70,043

Figure S2 presents the FTIR spectra of pure SBA-15, S_IL samples and its CeO_2_ nanocomposites. Characteristically, the signals assigned to SBA-15 vibrational modes were observed in all samples, with minor changes for the S_IL:Ce, indicating a slight interaction with silica material. The band around 1630 cm^–1^ is assigned to H–O–H bending vibration of H_2_O adsorbed. The asymmetric and symmetric vibrations of siloxane groups (Si–O–Si) appear at 1055 cm^–1^ (ν_as_ Si–O–Si), 965 cm^–1^ and 798 cm^–1^ ν_s_ (Si–O– Si), and the bending vibration δSi–O–Si is observed at 440 cm^–1^. In addition, the absence of signal around 1370 cm^–1^ due to the stretching vibration of the NO_3_^–^ groups proves the complete decomposition of nitrates present in the Ce precursor.^66,67^

The Raman spectra displayed in Figure 4 reveal a characteristic peak at 465 cm^–1^ in all S:IL:Ce samples. This single band is associated with the six degenerate active F_2g_ modes, characteristic of the fluorite-type cubic crystal structure with space group *Fm3m* of cerium oxide.^64,68,69^ This vibration mode arises from a symmetric axial deformation of the Ce–O bond, where, in the fluorite structure, the oxygen exhibits mobility while the adjacent cerium cations remain immobile.^70,71^ The spectrum for the sample S:Ce prepared in the absence of IL (Figure 4) presents the same band as the nanocomposites, confirming the presence of CeO_2_ with a fluorite-type cubic crystal structure. As expected, this band at 461 cm^–1^ is absent on the SBA-15 sample. It is possible to observe that this band at 461 cm^–1^ is narrower in the S_DMIBr:Ce and S_DMIBF4:Ce samples than in the S:Ce sample. This reflects a more organized crystalline structure of CeO_2_ crystallites in these samples prepared with ILs. The Raman spectroscopy results are consistent with the XRD findings discussed earlier.

**Figure 4:**
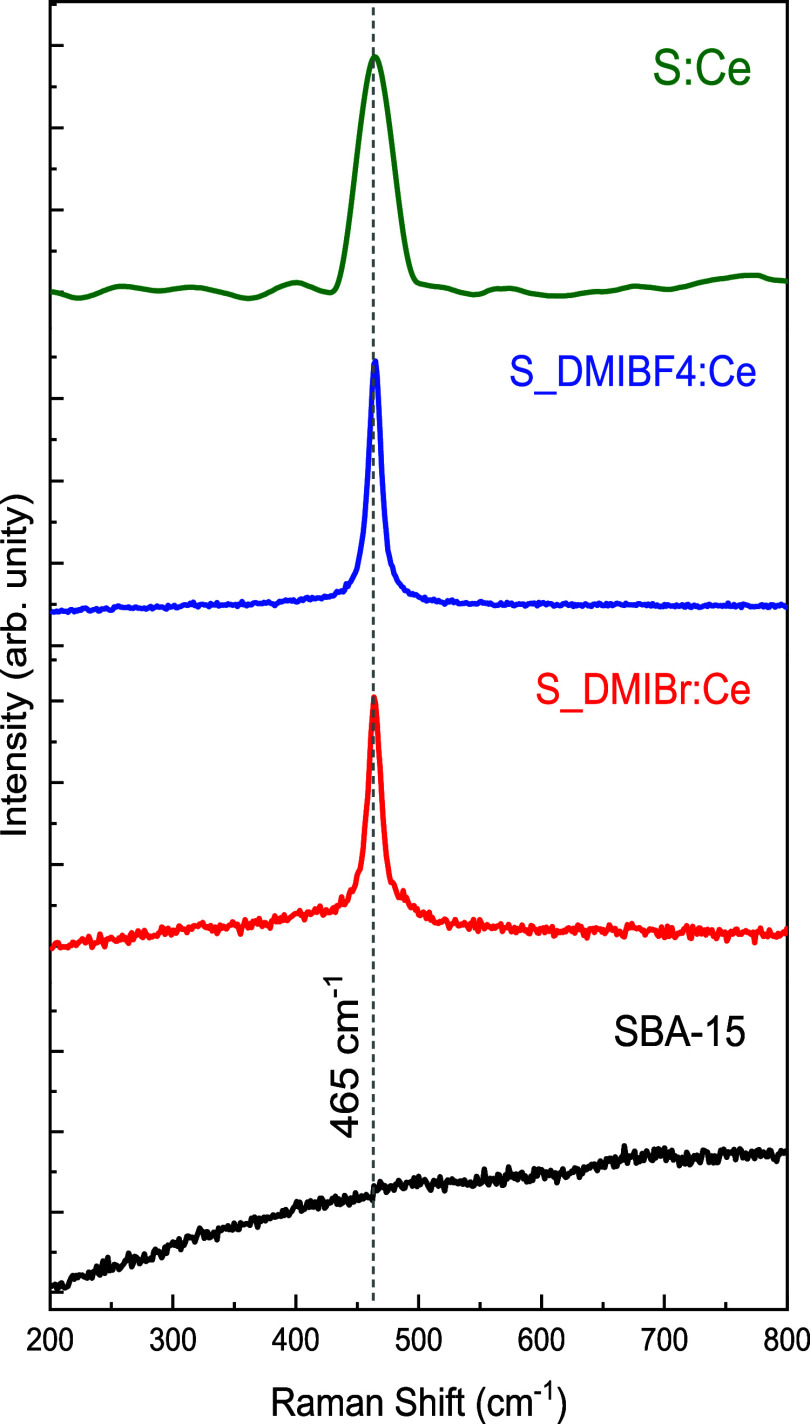
Raman spectra of SBA-15, S:Ce, S_DMIBr:Ce, and S_DMIBF_4_:Ce samples.

The N_2_ adsorption–desorption isotherms of SBA-15, S_LI, and S_LI:Ce samples are presented in Figure 5 (A1-B1), accompanied by their respective pore size distributions obtained from BJH adsorption isotherms (Figure 5, A2-B2). All isotherms are classified as type IV, according to the IUPAC classification,^72,73^ displaying H1 hysteresis loops characteristic of cylindrical mesoporous materials such as pure SBA-15.^56,74^ These results demonstrate that, regardless of the ionic liquids used in the synthesis, the formation of ordered mesoporous silica was achieved in all samples, as reported in previous work of our group,^31^ corroborating the SAXS data presented in Figure 2 and Table 1.

**Figure 5:**
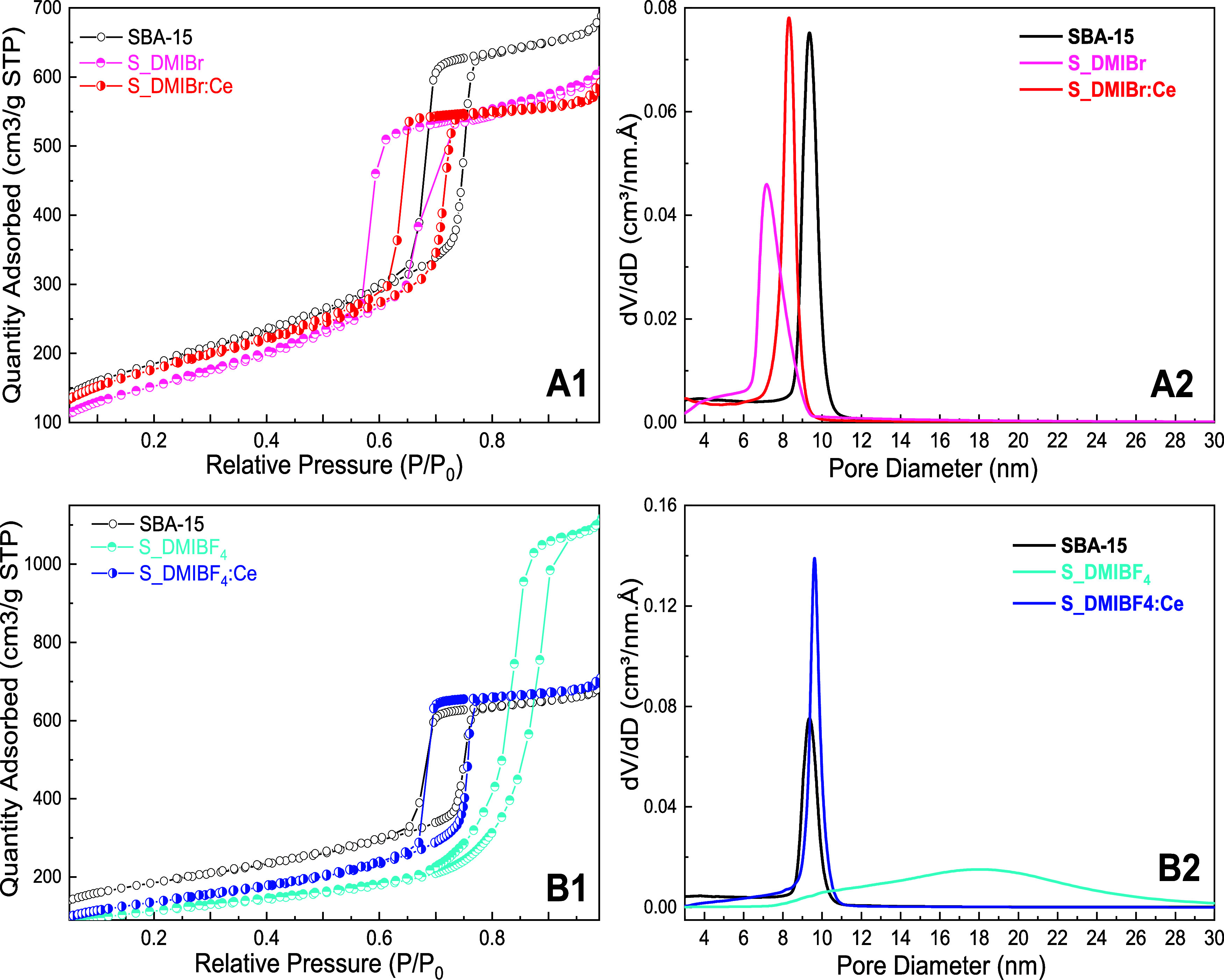
N_2_ adsorption–desorption isotherms (A1 and B1) and pore size distributions (A2 and B2) for the prepared materials: SBA-15, S_LI (S_DMIBF_4_, S_DMIBr), and S_LI:Ce (S_DMIBF_4_:Ce, S_DMIBr:Ce).

Table 3 presents the values of specific surface area (*S*_(BET)_), pore size (*D*_BJH‑adsorption_), and pore volume (*V*_p_), obtained from N_2_ adsorption–desorption Isotherm analysis. The S_LI sample prepared in BF_4_^–^-based ionic liquid exhibits a lower surface area and a more elongated hysteresis compared to its counterparts prepared with bromide anion, suggesting a larger extension of the pores. Also, it shows a substantial increase in pore diameter, also indicating changes in the pore structure. This may be related to a disturbance in the hydrophobic–hydrophilic equilibrium of Pluronic P123 micelles in the presence of this ionic liquid. The pore volumes of all samples are quite similar to those of SBA-15, except for the S_DMIBF_4_ sample, which has the highest pore diameter. Moreover, it is noted that the presence of CeO_2_ leads to an increase in the surface area when comparing the pair S_IL and S_IL:CeO_2_. This suggests that incorporating CeO_2_ into the mesoporous structure enhances the surface area, possibly due to the formation of additional active sites or changes in the pore walls’ morphology.

**Table 3:** Textural Properties of SBA-15 and SBA-15/CeO_2_ Nanocomposites Prepared with Different Ionic Liquids^a^

	N_2_ sorption isotherm data		
samples	*S*_(BET)_ (m^2^ g^–1^)	*D*_(BJH)_ (nm)	*V*_p_ (cm^3^ g^–1^)
SBA-15	665	9.4	1.5
S_DMIBr	549	7.0	1.3
S_DMIBF_4_	409	17.3	1.9
S_DMIBr:Ce	636	8.3	1.4
S_DMIBF_4_:Ce	491	9.6	1.4

a *S* = surface area; BET = Brunauer–Emmett–Teller, BJH = Barrett–Joyner–Halenda; *D* = pore diameter; *V*_p_ = pore volume.

The N_2_ physisorption isotherm for the sample prepared without IL is presented in Figure S3 of the Supporting Information. The result confirms the absence of mesostructure, indicating the collapse of the material without the presence of IL, as previously shown by the SAXS results in Figure S1.

Figure 6 presents the SEM images of SBA-15 and S_IL materials. The SBA-15 particles display a rod-shaped morphology with a size of approximately 1 μm. When prepared in DMIBF_4_, the particles maintain a similar rod-like morphology with sizes ranging between 1–2 μm. In contrast, in the presence of Br-based ionic liquid, the surface morphology and the particles’ size and shape change significantly, resulting in the formation of a disordered morphology with agglomerated SBA-15 particles. These data indicate that the presence of bromide-based ionic liquids significantly impacts the final morphology and surface characteristics of the materials. Figure 7 exhibits images of S_IL:Ce nanocomposites prepared in both ionic liquids. The particle morphology differs slight from the corresponding S_IL samples, indicating that the cerium source also influences the silica particle morphology. While the S_DMIBF_4_:Ce sample exhibits rod-shaped particles similar to their S_IL counterparts, the S_DMIBr:Ce nanocomposite exhibits more spherical particles. Additionally, the presence of needle-shaped particles outside the larger particles is noticeable at a high magnification (10,000×). These needle-shaped CeO_2_ particles are likely ceria nanoparticles formed outside the mesopores, in agreement with XRD and NAI results.

**Figure 6:**
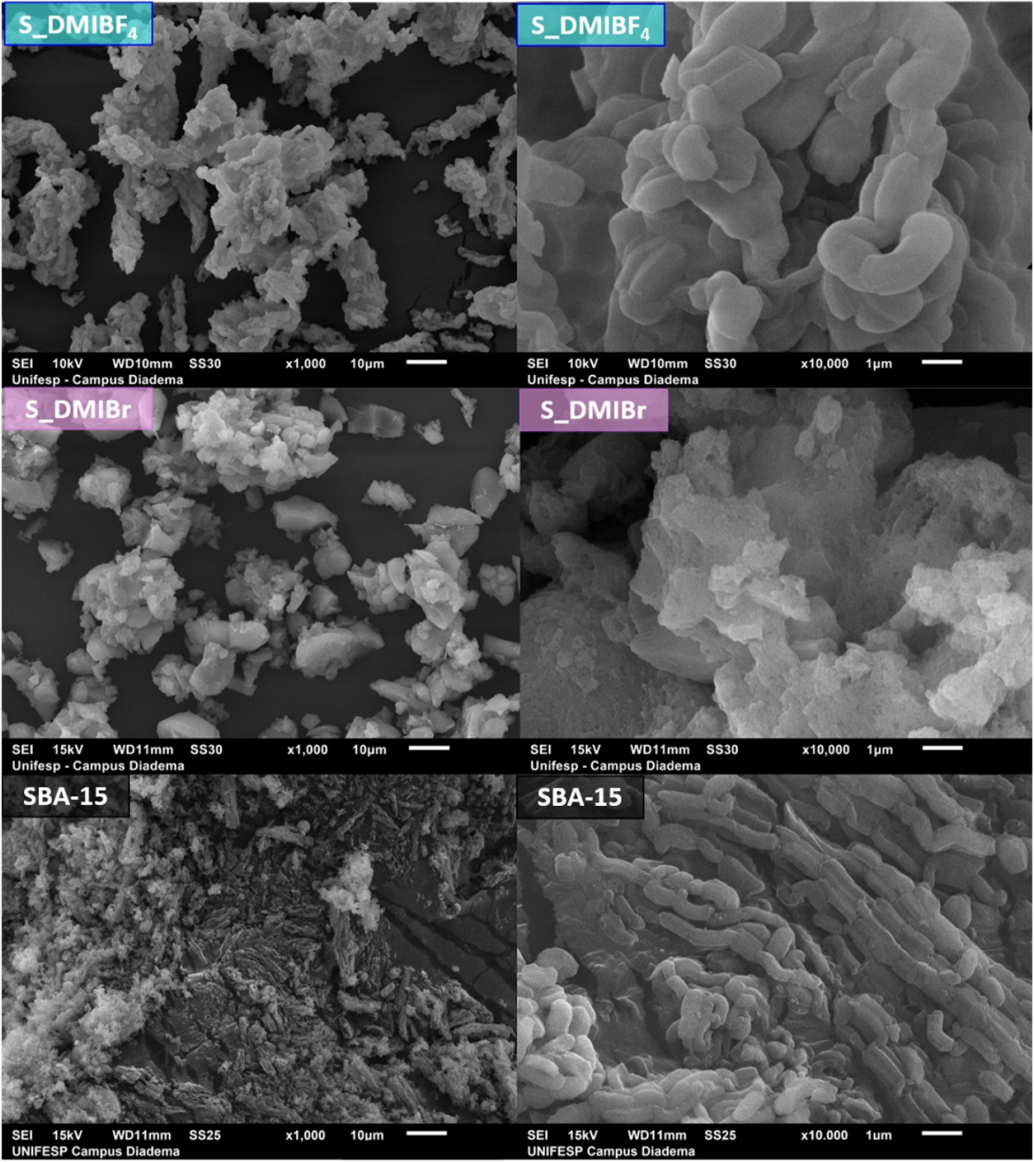
SEM images of the SBA-15, S_DMIBr, and S_DMIBF_4_ samples.

**Figure 7:**
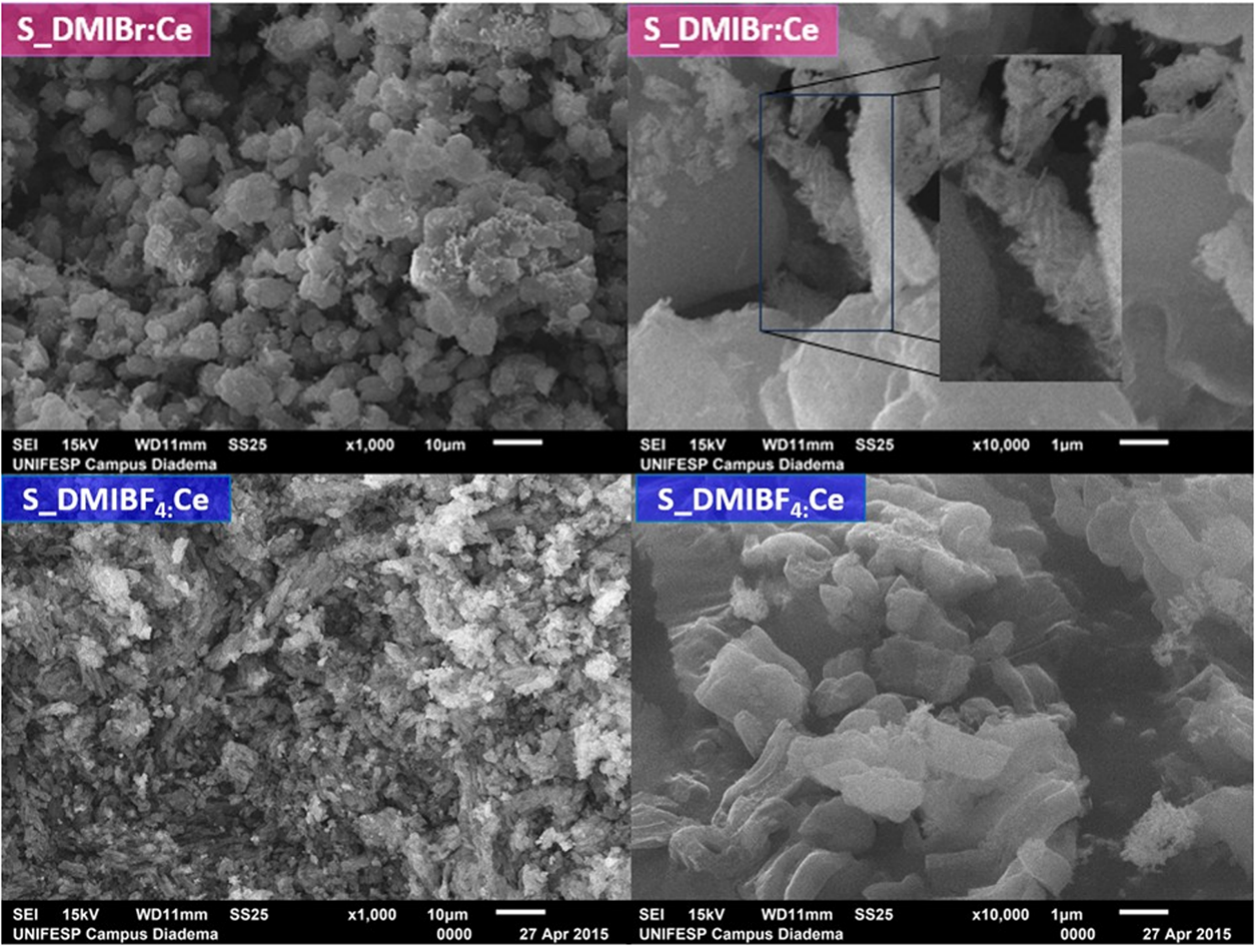
SEM images of the S_DMIBr:Ce and S_DMIBF_4_:Ce samples.

Figure 8 shows the XPS spectra of the S_LI: Ce nanocomposites. Figure 8A displays the Si 2p results in the band energy range between 95 and 110 eV, with the Si 2p_3/2_ signal between 102 and 108 eV corresponding to Si of silanol and siloxane groups of SBA-15.^75^ Figure 8B corresponds to the O 1s spectra in the range from 525 to 545 eV. The more intense band, around 534 eV, is assigned to oxygen from Si–OH, Si–O–Si species, and adsorbed water.^75,76^ The band at 530 eV indicates the surface and lattice of O^2–^ species in CeO_2_ or SiO_2_.^23,76,77^

**Figure 8:**
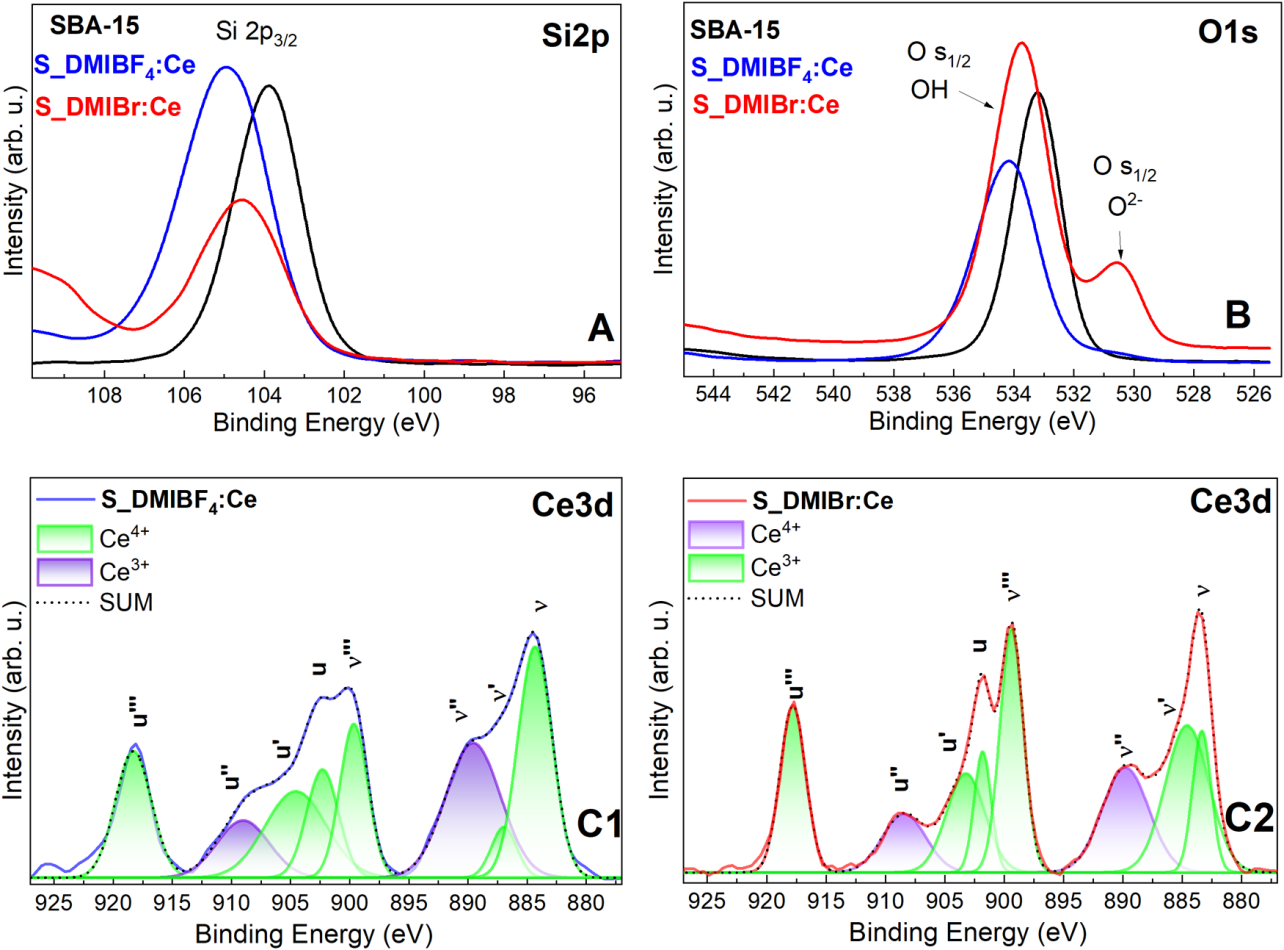
XPS spectra of (A) Si 2p, (B) O 1s, and (C) Ce 3d for the S_LI:Ce nanocomposites.

The Ce 3d spectra for the four S_LI: Ce nanocomposites are shown in Figure 8C1–C2. The eight peaks were deconvoluted using Lorentzian and are attributed to Ce 3d_5/2_ and Ce 3d_3/2_. Therefore, the nanocomposites contain both oxidation states Ce^3+^ and Ce^4+^. For Ce 3d_5/2_, the peaks at 882.1, 884.1, 888.4, and 897.5 eV are presented in spectra as v, v′, v″, and v″, respectively. For Ce 3d_3/2_, the peaks at 901, 904, 908, and 919.6 eV are presented as u, you′, u″, and u″, respectively. The you‴, u″, u, v‴, v″ and v peaks are attributed to Ce^4+^ ions, while u′ and v′ are characteristic of Ce^3+^ ions.^76–79^ The presence of Ce^3+^ is assigned to the presence of oxygen defects in the crystalline structure of CeO_2._ The presence of these defects is important as they serve as active centers for catalytic processes, enhancing the material’s effectiveness in such applications.^23^

The TGA and DSC curves of the different prepared materials (SBA-15, S_LI, and S_LI:Ce) are illustrated in Figure 9A,B. For all samples, the weight loss observed in the temperature range of 30–120 °C, accompanied by an endothermic peak around 60 °C in DSC curves, is attributed to the elimination of physically adsorbed water from the material’s surface (Table 4). The second event, occurring between 120 and 900 °C, corresponds to the condensation of silanol groups on the SBA-15 surface, accompanied by an exothermic peak in DSC curves. The S_LI samples prepared with Br^–^-based ionic liquid exhibit a higher value of adsorbed water, around 5%, than those prepared in BF_4_^–^-based ionic liquid, which present 1.5%. The samples containing CeO_2_ particles show an increased amount of adsorbed water: 10.9% for S_DMIBr:Ce and 4.1% for those prepared with BF_4_^–^-based nanocomposites. These results can be attributed to better structuration for materials synthesized with the Br^–^ anion. Additionally, the presence of CeO_2_ nanoparticles once again demonstrates its influence on the structural properties, as observed by SAXS and NAI, resulting in more physisorption sites.

**Figure 9:**
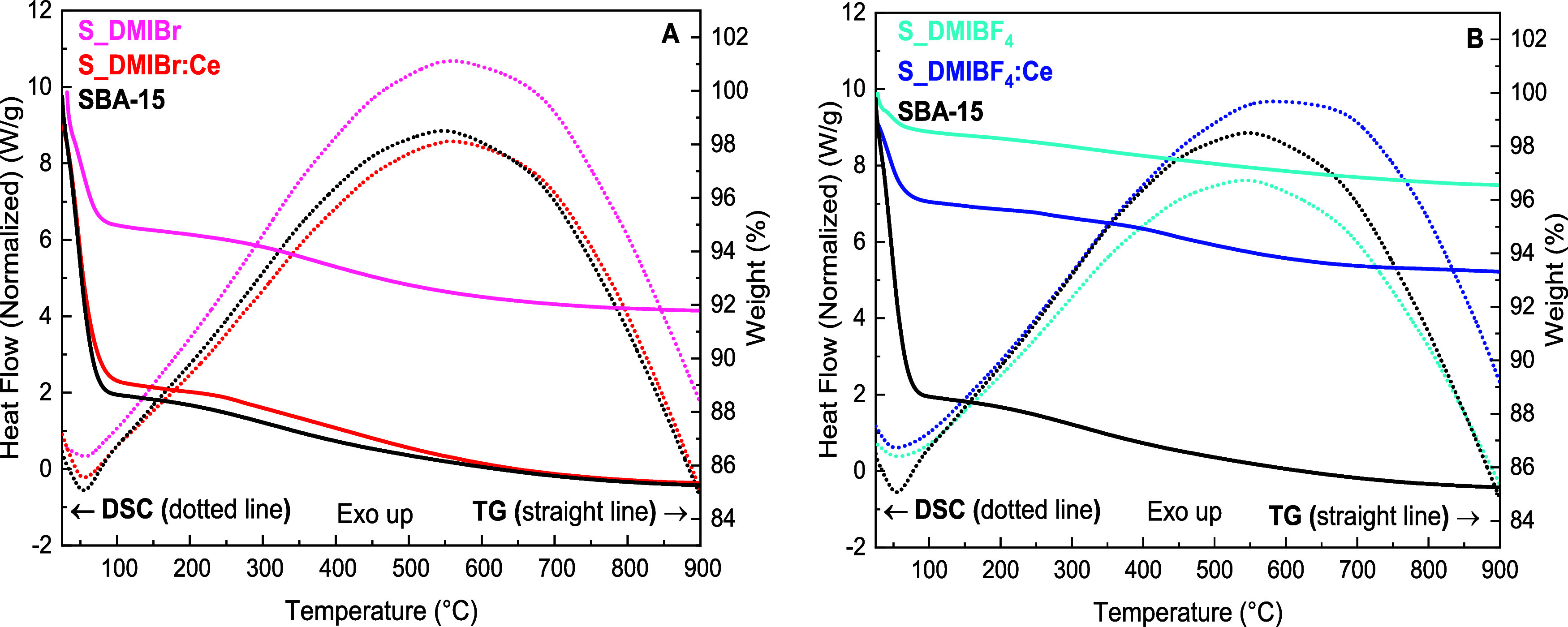
TGA/DSC curves of (A) SBA-15, S_DMIBr, and S_DMIBr:Ce, and (B) SBA-15, S_DMIBF_4_, and S_DMIBF_4_:Ce.

**Table 4:** TGA Analysis of SBA-15 and SBA-15/CeO_2_ Nanocomposites Prepared with Different Ionic Liquids^a^

	first event 25–120 °C		second event 120–1000 °C		
samples	Δwt (%)	*T*_onset_ (°C)	Δwt (%)	*T*_onset_ (°C)	*R* (%)
SBA-15	11.6	25.5	3.1	259.2	85.2
S_DMIBr	5.3	32.8	3.1	279.0	91.8
S_DMIBF_4_	1.5	28.9	2.0	241.9	96.4
S_DMIBr:Ce	10.9	22.4	3.7	241.5	85.3
S_DMIBF_4_:Ce	4.1	20.8	2.6	348.7	93.2

a *T*_onset_ = extrapolated onset temperature, Δ*w* = weight loss, *R* = residue.

Figure 10 shows the TEM images for S_DMIBr, S_DMIBF_4,_ S_DMIBr:Ce, and S_DMIBF_4_:Ce samples. The mesoporous channels characteristic of SBA-15 are visible in the micrographs, regardless of the anion employed in the synthesis. The well-ordered hexagonal arrangement of the pores is distinctly highlighted, with the regular pore spacing emphasized with rectangular markers. These findings are consistent with the SAXS data, which show reflections corresponding to the two-dimensional ordering of the mesopores. To confirm the dispersion of CeO_2_ within the matrix, Energy-Dispersive X-ray Spectroscopy (EDS) analysis was performed on the Ce-based nanocomposites. The specific location analyzed by EDS was marked with a dotted circle in Figure 10. The corresponding EDS spectra, displayed in Figure 11, affirm the presence of Ce nanoparticles within the nanocomposite structure, supporting the successful incorporation of CeO_2_ into the matrix.

**Figure 10:**
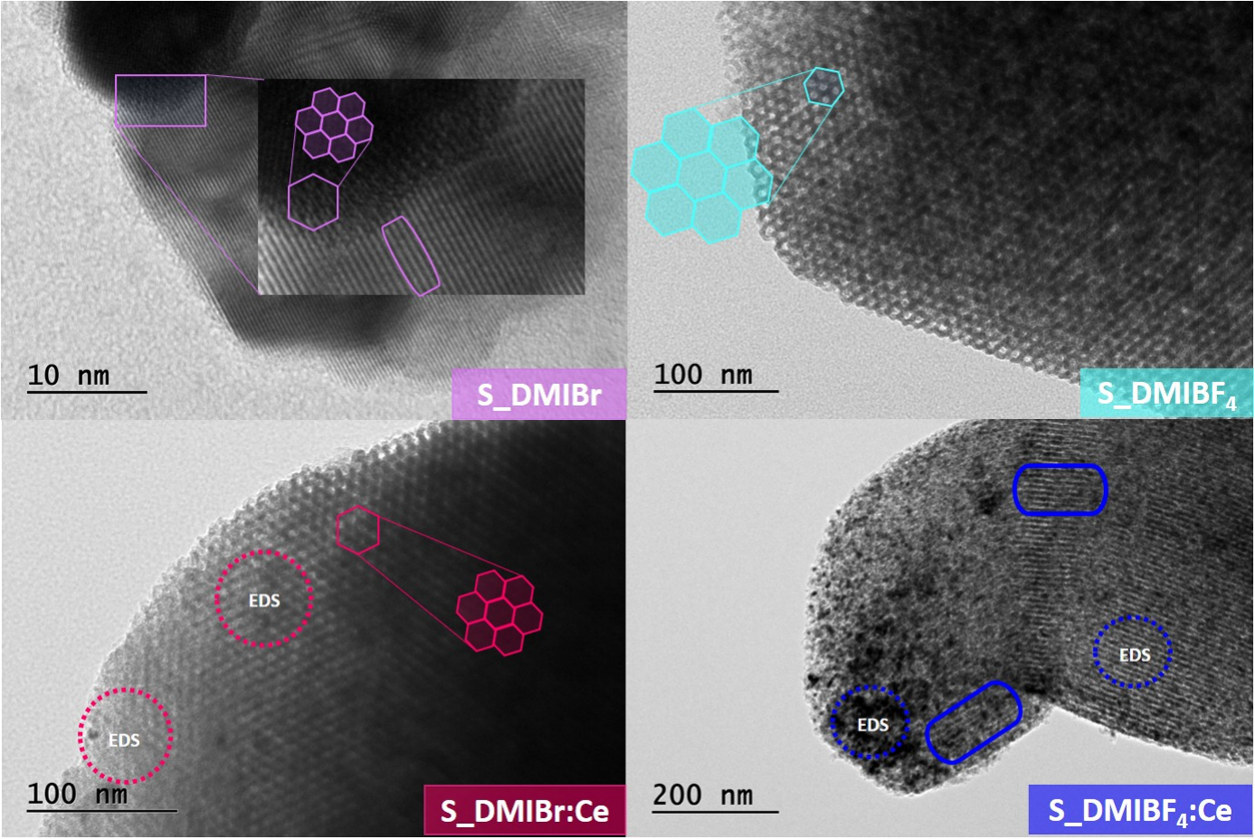
Transmission Electron Microscopy (TEM) analysis illustrating the ordered channels and hexagonal mesoporous structures of S_DMIBr, S_DMIBr:Ce, S_DMIBF_4_, and S_DMIBF_4_:Ce nanocomposites. The dotted circle highlights the specific region analyzed via EDS, with corresponding spectra presented in Figure 11.

**Figure 11:**
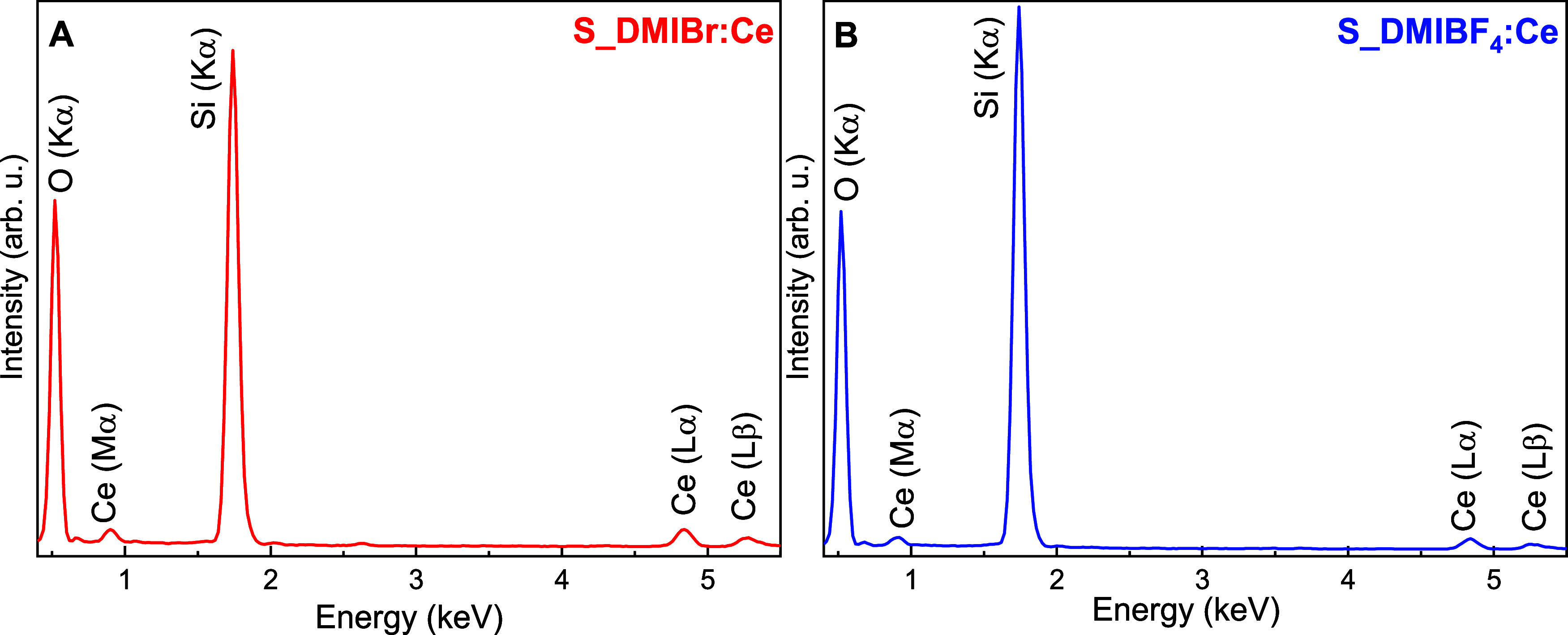
EDS spectra of nanocomposites (A) S_DMIBr:Ce and (B) S_DMIBF_4_:Ce.

The EDS spectra (Figure 11A,B) for both nanocomposites (S_DMIBr:Ce and S_DMIBF_4_:Ce) reveal prominent peaks for silicon (Si Kα) at approximately 1.7 keV and oxygen (O Kα) at 0.5 keV, indicating a silica-based matrix. Additionally, peaks associated with cerium (Ce) are clearly observed, including Ce (Mα) around 0.9 keV, Ce (Lα) near 4.8 keV, and Ce (Lβ) at approximately 5.3 keV, confirming the successful incorporation of cerium into the nanocomposite structure.

### 3.1 Influence of DMIBr and DMIBF_4_ in Synthesis

Incorporating ionic liquids (ILs) during the synthesis of SBA-15 mesoporous silica significantly influences the structural properties of the resulting materials. In this study, SBA-15 was synthesized using both the conventional method and in the presence of two different ILs: 1,3-dimethylimidazolium bromide (DMIBr) and 1,3-dimethylimidazolium tetrafluoroborate (DMIBF_4_). These ILs, with their distinct anionic species, affect the mesostructure, pore size distribution, and overall ordering of the SBA-15 framework, primarily due to their specific interactions with the surfactant micelles.

DMIBF_4_, a more hydrophobic ionic liquid, tends to localize preferentially within the hydrophobic core of the P123 surfactant micelles at low concentrations.^80^ This positioning causes swelling of the micelle due to increased hydrophobic interactions, resulting in changes in the sizes of the micelle that translate into different pore diameters in the synthesized mesoporous materials. Since DMIBF_4_ does not significantly interact with the hydrophilic poly­(ethylene oxide) (PEO) corona of the micelles at these concentrations, minimal dehydration of the corona occurs, allowing the micelle to maintain an expanded state in relation to DMIBr. However, the resulting larger micelle size and reduced interaction with the corona can disrupt the close packing of micelles, leading to a broader pore size distribution and decreased structural ordering in the final material.^80,81^

In contrast, DMIBr, which contains the smaller anion, is a more hydrophilic ionic liquid and interacts not only with the hydrophobic core but also with the hydrophilic PEO corona of the micelles.^82^ This interaction promotes dehydration of the PEO corona, which leads to micelle contraction and a closer packing arrangement.^82,83^ The contracted micelles contribute to a highly ordered hexagonal mesophase, resulting in a narrower pore size distribution and smaller pore diameters in the SBA-15 synthesized with DMIBr.

It is worth noting that the effects of ILs on micelle behavior are concentration-dependent. Literature reports indicate that, at higher IL concentrations, even hydrophobic anions like BF_4_^–^ can induce micelle contraction by interacting with both the core and corona, leading to dehydration of the hydrophilic segments.^80,84–86^ However, in this study, the IL concentration was kept low, allowing us to observe the preferential localization of DMIBF_4_ in the micelle core and the swelling effect without significant corona dehydration.

The effect of ILs on the synthesis of SBA-15/CeO_2_ nanocomposites was distinct, since the differences between the two ILs became less pronounced in the structural properties of the resulting materials (S_DMIBr: Ce vs S_DMIBF_4_:Ce). This observation suggests that the cerium ions play a dominant role in the micelle structuring process, potentially dominating the distinct effects of the IL anions. The “salting in” effect of Ce­(NO_3_)_3_·6H_2_O, where Ce^3+^ ions interact electrostatically with the Pluronic P123 surfactant, promotes micelle expansion, particularly at the hydrophilic interface.^87^ This effect causes swelling of the micelle corona and leads to larger pore diameters in the final material, regardless of the specific IL used. The cerium ions preferentially interact with the PEO corona, pushing the ILs toward the hydrophobic core, and exerting a secondary effect.^88^ Consequently, the presence of cerium governs the formation and stability of the mesoporous structure, while the IL primarily helps stabilize the micelle core. In conclusion, the superior structuring of the composites, compared to the silicas synthesized with the corresponding ILs, can be attributed to the higher capability of Ce^3+^ and NO_3_^–^ ions to form ion–water complexes. This interaction enhances the micellization of the PEO–PPO-PEO block copolymer more efficiently in aqueous solution. A proposed scheme illustrating the mechanisms of interaction between ILs and cerium precursor on the SBA-15 structure is presented in Figure 12.

**Figure 12:**
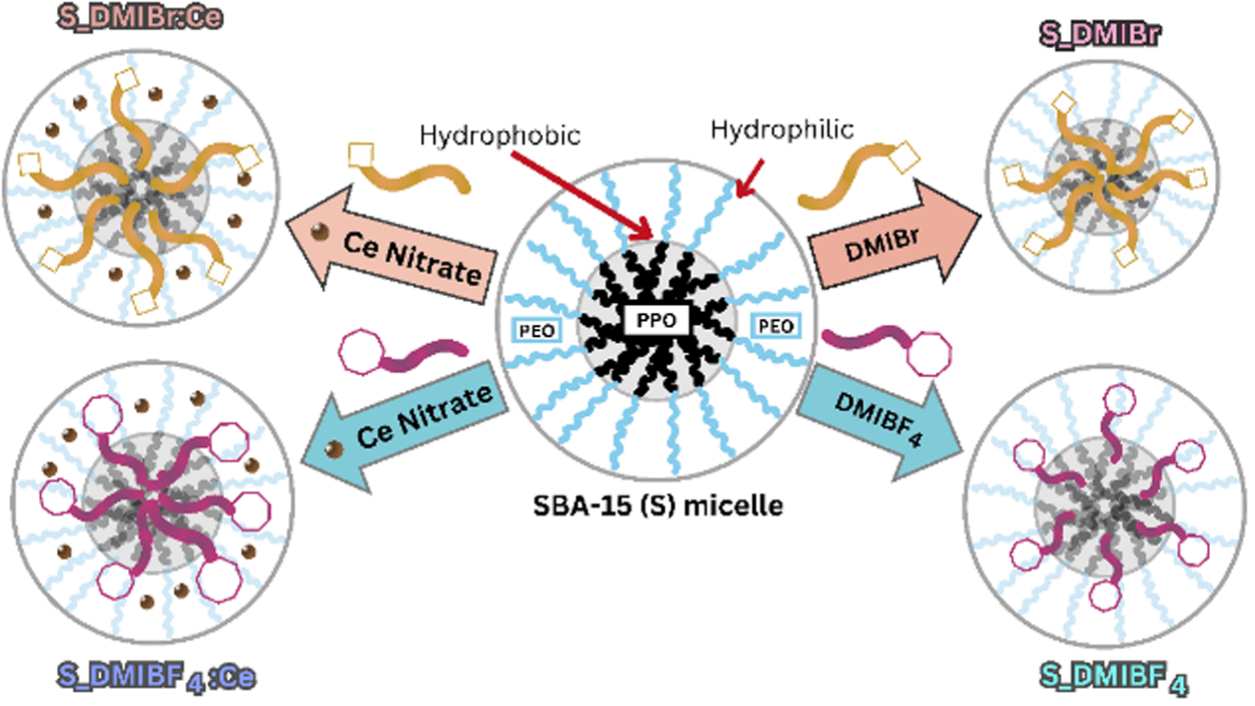
Schematic representation of the interaction mechanisms between Pluronic P123, ionic liquids, and the cerium precursor on the SBA-15 structure.

## 4 Conclusions

This study explored the use of two distinct ionic liquids (DMIBr and DMIBF_4_) in the synthesis of SBA-15 and SBA-15:CeO_2_ nanocomposites. The objective was to investigate how variations in the anionic components (bromide versus tetrafluoroborate) influence the overall structural and morphological characteristics of the final materials. The results show that, despite the inclusion of CeO_2_, the typical ordered mesoporous structure of SBA-15 is maintained. However, the type of ionic liquid used plays a critical role in determining pore organization and dimensions. In particular, the bromide-based ionic liquid promotes a more uniform and well-organized mesostructure compared to its tetrafluoroborate counterpart, which leads to larger and more variable pore sizes. Furthermore, the integration of CeO_2_ appears to enhance the ordering and may increase the availability of adsorption sites. These findings underline the significant impact that the anionic component of the ionic liquid has on tailoring the textural and morphological properties of SBA-15:CeO_2_ nanocomposites, offering valuable insights for applications in environmental remediation and other fields where precise material properties are essential.

## Supplementary Material



## Notes

Additional information regarding the results cited throughout the manuscript, such as the data of the amount of reagent used to prepare the materials studied in this work (Table S1), the unstructured SAXS curve of S_Ce prepared in the absence of IL (Figure S1), the FTIR spectra for SBA-15, S_IL and S_IL:Ce (Figure S2), and the nitrogen physisorption isotherms for unstructured sample S_Ce (Figure S3) (PDF)
