# Supplementary material for: Effect of Ionic Liquids on the Structural Properties of SBA-15/CeO2 Nanocomposites
Source: ACS Omega. 2025 May 27;10(22):23374–86. doi: 10.1021/acsomega.5c01826 (PMC12163833; doi:10.1021/acsomega.5c01826)
Supplement: Supplementary file 1 [file ao5c01826_si_001.pdf]

Supporting Information

Effect of ionic liquids on the structural properties of SBA-15/CeO_2 nanocomposites

Danilo W. Losito[a], Renato M. Latini[a], Norberto S. Gonçalves[a], Fernanda F. Camilo[a], Márcia C. A. Fantini[b], Tereza S. Martins*[a]

[a]Departamento de Química, Instituto de Ciências Ambientais, Químicas e Farmacêuticas, Universidade Federal de São Paulo, Diadema, SP, Brazil

[b]Laboratório de Cristalografia, Instituto de Física, Universidade de São Paulo, São Paulo, SP, Brazil

*corresponding author

Email: tsmartins@unifesp.br

Additional information of the results, cited throughout the manuscript, such as the data of the amount of reagent used to prepare the materials studied in this work (Table S1), the unstructured SAXS curve of S_Ce prepared in the absence of IL (Figure S1), the FTIR spectra for SBA-15, S_IL and S_IL:Ce (Figure S2), and the isotherm of nitrogen physisorption for unstructured sample S_Ce (Figure S3).

Table S1. Quantity of the reagents used in the preparations

	Quantity			
**Material**	**Pluronic P123 (g)**	**TEOS (g)**	**Ionic Liquid (mol)**	**$Ce(NO_3)_3.6H_2O$ (g)**
SBA-15	2.00	4.20	----	----
S:Ce	2.00	4.20	----	0.93
S_IL	2.00	4.20	$7{,}0 \times 10^{-4}$	----
S_IL:Ce	2.00	4.20	$7{,}0 \times 10^{-4}$	0.93

The amount of IL is different for each synthesis, depending on the molecule used., following the molar mass below: DMIBr = 330.9 g mol^{-1}. $DMIBF_4$= 337.76 g mol^{-1}.

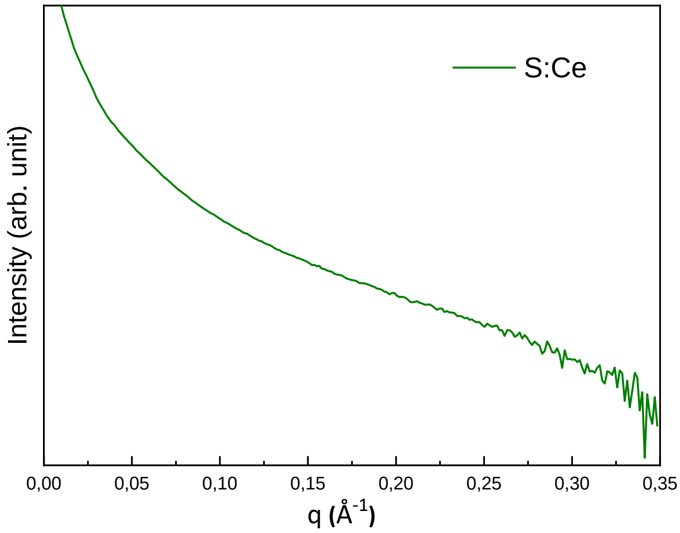

Figure S1. SAXS curve for the nanocomposite prepared by direct synthesis, without IL in the reaction medium, S:Ce. The result shows the absence of the five characteristic peaks of a mesostructured of the SBA-15.

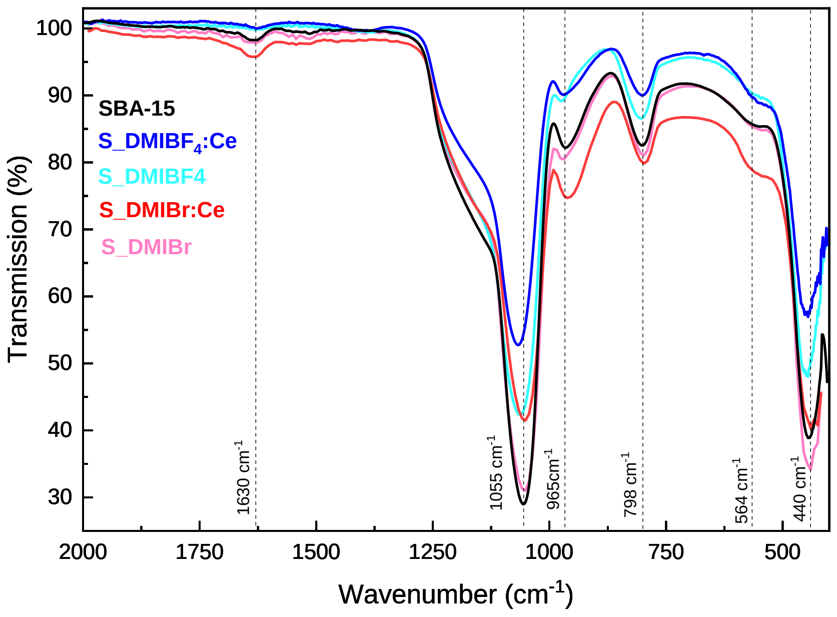

Figure S2. FTIR spectroscopy for the SBA-15, S_IL and S_IL:Ce.

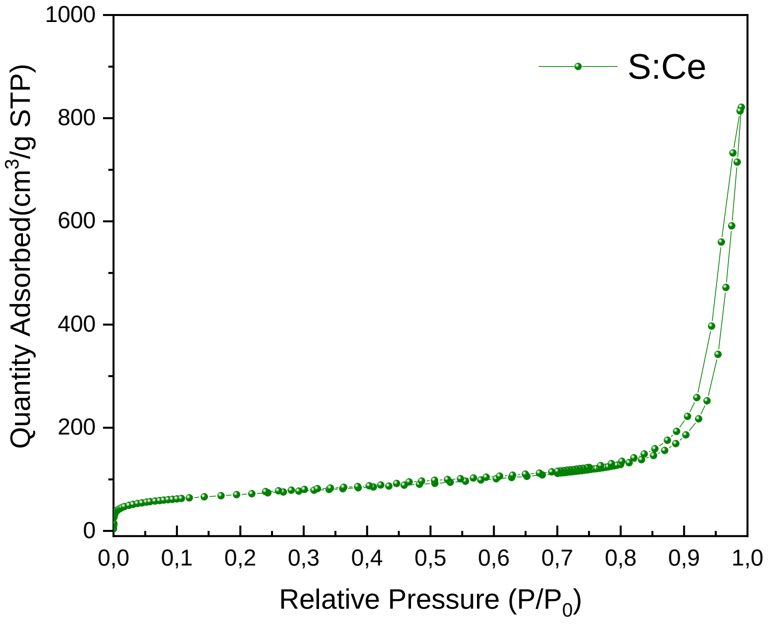

Figure S3. N_2 physisorption isotherm for sample S:Ce. The absence of type 2 hysteresis confirms that there is no mesoporous structure.
